# A prospective cohort study to assess the micro-epidemiology of *Plasmodium falciparum* clinical malaria in Ilha Josina Machel (Manhiça, Mozambique)

**DOI:** 10.1186/s12936-016-1496-y

**Published:** 2016-08-31

**Authors:** Beatriz Galatas, Caterina Guinovart, Quique Bassat, John J. Aponte, Lídia Nhamússua, Eusebio Macete, Francisco Saúte, Pedro Alonso, Pedro Aide

**Affiliations:** 1 Centro de Investigação em Saúde de Manhiça (CISM), Rua12, Bairro Cambeve, Vila de Manhiça, Maputo Mozambique; 2 ISGlobal, Barcelona Ctr. Int. Health Res. (CRESIB), Hospital Clínic-Universitat de Barcelona, Barcelona, Spain; 3 Global Malaria Programme, World Health Organization, Geneva, Switzerland; 4 National Institute of Health, Ministry of Health, Maputo, Mozambique

**Keywords:** Clinical malaria, *Plasmodium falciparum*, Epidemiology, Transmission, Incidence, Mozambique

## Abstract

**Background:**

After the decrease in clinical malaria incidence observed in Mozambique until 2009, a steady resurgence of cases per year has been reported nationally, reaching alarming levels in 2014. However, little is known about the clinical profile of the cases presented, or the possible epidemiological factors contributing to the resurgence of cases.

**Methods:**

An analysis of surveillance data collected between July 2003 and June 2013 in the high malaria-transmission area of Ilha Josina Machel (Southern Mozambique) through a paediatric outpatient morbidity surveillance system was conducted to calculate hospital-based clinical malaria rates, slide-positivity rates, and minimum community-based incidence rates (MCBIRs) and incidence rate ratios per malaria season in children younger than 15 years of age. Clinical malaria was defined as a fever ≥37.5 °C or a reported fever in the previous 24 h with a positive blood smear. Yearly mean age, geometric mean parasitaemia (GMP) and mean packed cell volume (PCV) were also described for all clinical malaria cases and compared between seasons using DID analysis or ANOVA tests.

**Results:**

During the study period, the percentage of outpatient visits presenting with confirmed clinical malaria decreased from 51 % in the 2003–2004 season to 23 % in 2008–2009, followed by an increase back to 51 % in 2012–2013. The yearly mean age of cases significantly increased from 2.9 (95 % CI 2.8–3.0) in 2003–2004 to 5.7 (95 % CI 5.6–5.7) in 2012–2013, compared to non-malaria cases. An increase in mean PCV levels was also observed (p < 0.001), as well as in GMPs: from 5778 parasites/µL in 2002–2003 to 17,316 parasites/µL in 2012–2013 (p < 0.001) mainly driven by an increase in GMP in children older than 1 year of age. MCBIRs in infants decreased by 70 % (RR = 0.3, p < 0.001) between 2003–2004 and 2012–2013. Incidence diminished by a third among children 1- to 4-years between 2003 and 2007, although such drop was unsustained as observed in 2012–2013 (RR = 1.0, 95 % CI 0.9–1.0). Finally, the incidence among children 5–14 years was 3.8 (95 % CI 3.4–4.3) times higher in 2012–2013 compared to 2003–2004.

**Conclusion:**

Since 2003, Ilha Josina Machel observed a significant reduction of clinical malaria cases which was followed by an upsurge, following the national trend. A shift in the age distribution towards older children was observed, indicating that the changes in the transmission intensity patterns resulted in a slower acquisition of the naturally acquired immunity to malaria in children.

## Background

As part of the Millennium Development Goals (MDGs) established by the United Nations in the year 2000, all malaria-endemic countries concentrated their efforts on reducing malaria incidence globally by 2015. In the last decade, 55 of the 106 countries with ongoing malaria transmission reached the target set in Objective 6 to reduce their malaria burden by 75 %. However, these countries contributed only to 6 % of the global estimated cases, suggesting that countries with the most affected populations, most of them from sub-Saharan Africa, have yet to reach this target [1].

Mozambique is part of the ten countries with the highest malaria endemicity in the world. Transmission intensity varies widely within the country, with high transmission intensity occurring in the north and a decreasing pattern observed as provinces are closer to the south of the country [2–4]. However, high transmission hotspots can still be found within wider low transmission areas. Despite the increasing funding available for malaria control, Mozambique has experienced an increase in malaria incidence since 2007, according to national malaria surveillance data [1]. Although it is not clear what the main factors are driving these increasing malaria incidence trends, coverage of malaria control interventions is heterogeneous, and interventions are not delivered on a regular basis [2, 5]. Indoor residual spraying (IRS) was first introduced in urban areas in 1994, whereby only ten districts in the country were selected. Insecticide-treated nets (ITN) deployment only started in 2004, primarily through donor funding. The south of Mozambique also took part in the Lubombo Spatial Development Initiative (LSDI), a tri-lateral initiative between the governments of Swaziland, South Africa and Mozambique that took place in the early 2000s, and officially finished its activities in 2011 [6, 7].

Monitoring malaria trends in the different areas of the country and characterizing changes in clinical presentation and age distribution of malaria cases during shifts in transmission is crucial to inform policy. High-quality, health facility-based surveillance data, despite the potential selection bias due to differences in health-seeking behaviour and accessibility, allow finely characterizing of clinical malaria epidemiology of an area over time, offering accurate and detailed information that can complement information on trends observed through the national health management information system. Data from the Manhiça Health Research Centre (CISM) outpatient paediatric morbidity surveillance system, operating in the southernmost province of Mozambique, identified heterogeneous epidemiological patterns within its study area. This study aims to analyse the epidemiological trends of the outpatient clinical malaria cases from 2003 until 2013 in an area of high endemicity within the district if Manhiça, that experienced a drop in transmission after IRS, ITNs, and artemisinin combination therapy (ACT), followed by a later resurgence of clinical cases. Evidence from this analysis may offer the National Malaria Control Programme (NMCP) additional insight on the clinical malaria profile that could be observed in other areas of the country that have experienced similar changes in malaria transmission.

## Methods

### Study design

This is an analysis of surveillance data collected through the Ilha Josina Machel health post (IJMHP) outpatient paediatric morbidity surveillance system. All visits in children younger than 15 years old from 1 July 2003 to 30 June 2013 were included.

### Study area and population

**Fig. 1:**
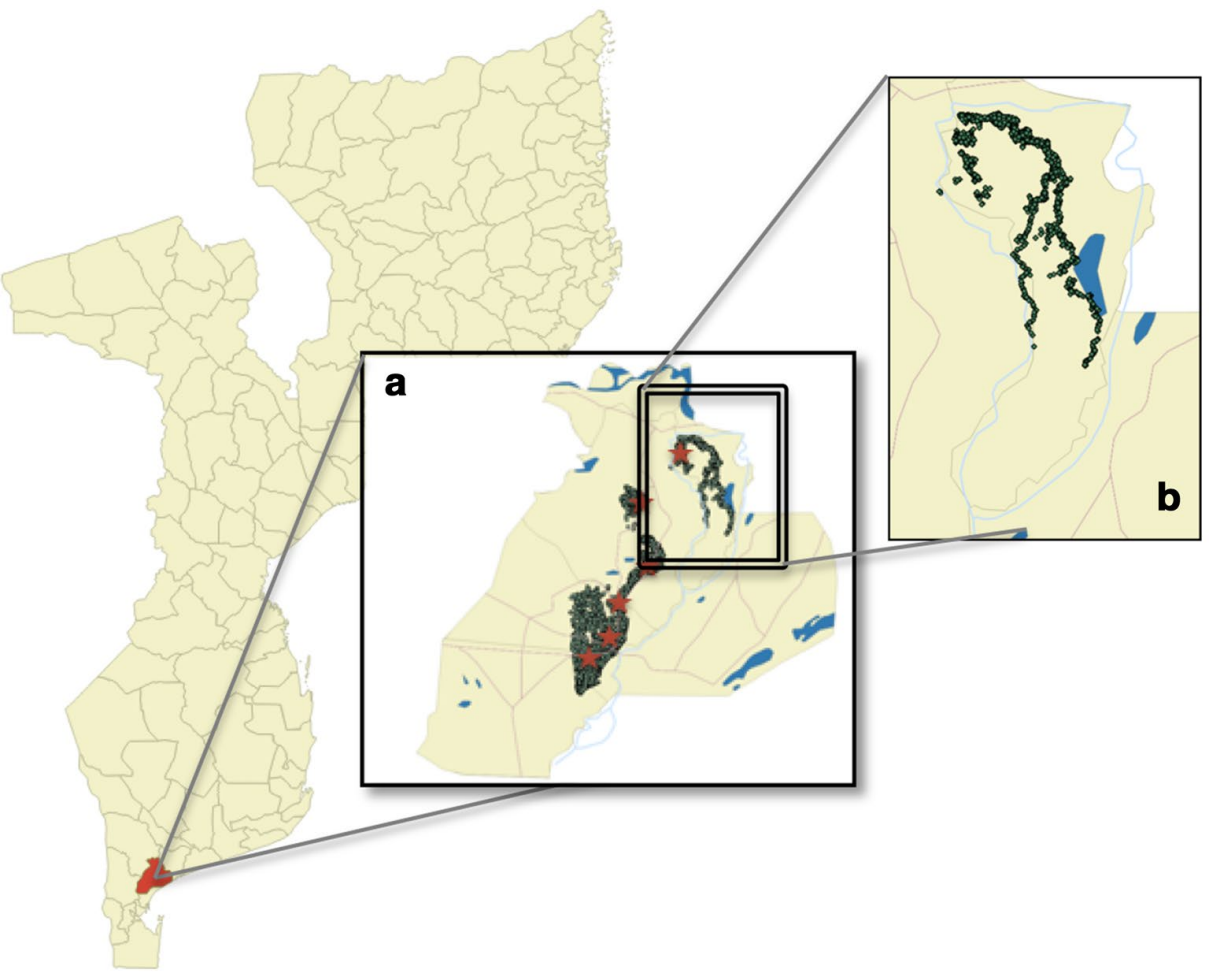
Maps of Mozambique, Manhiça and Ilha Josina. Ilha Josina Machel forms part of the Manhiça district, located in the South Mozambique. *Map a* represents the health facility catchment areas covered by CISM’s Health and Demographic Surveillance System, with the households in * green* and health facilities in * red*. *Map b* shows the IJMHP catchment area

Ilha Josina Machel is a 188 km^2^ river island in the confluences of the Incomati River, located about 50 km northeast of Manhiça village (Manhiça District, Maputo Province, Southern Mozambique) (Fig. 1). Approximately 15,000 people live currently in the area, 40 % of the population is under the age of 15 years, and 55 % are female. A third of the population is illiterate and the main occupation consists of subsistence agriculture. Houses are simple, with walls typically made of cane with thatched or corrugated roofs. Basic and preventive care is provided at the IJMHP, including antenatal and delivery services, but no admission facilities exist for sick patients, which are referred to Manhiça District Hospital in case of need. Additionally, as part of CISM’s study area, Ilha Josina Machel is covered by a demographic surveillance system (DSS), explained in detailed elsewhere [8], that identifies all residents within the catchment area and provides accurate yearly-updated estimates of community denominators.

**Fig. 2:**
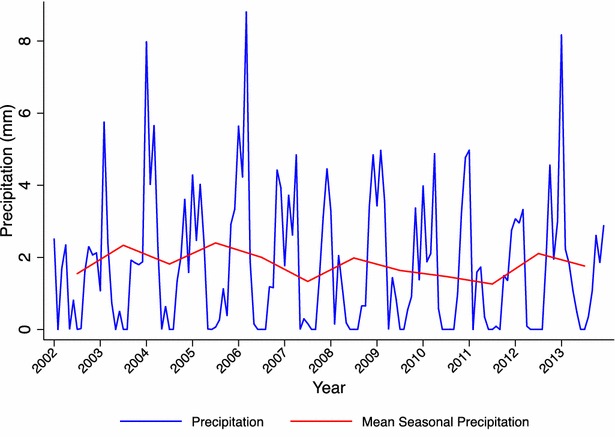
Monthly and seasonal precipitation (mm) in Manhiça Dictrict (2003–2013). Monthly rainfall data for every year in the study were accessed from the Climate Hazards Group InfraRed Precipitation with Station dataset [33]. Data were withdrawn for the coordinates of the district of Manhiça to obtain robust rainfall estimates. Mean rainfall estimates were calculated for every malaria season and compared through the study period

The region has two distinct seasons: a warm and rainy season from November to April, and a cooler and drier season the rest of the year. Rainfall trends varied throughout the study period, with wetter rainy seasons between 2002 and 2006, and slightly drier seasons between 2007 and 2012 (Fig. 2). Malaria transmission is perennial with seasonality, mostly due to *Plasmodium falciparum*, with a few sporadic cases of *Plasmodium malariae*, and with *Anopheles funestus* as the main vector. Malaria transmission was high in Ilha Josina Machel at the beginning of the study period. A cohort study to assess the *P. falciparum* re-infection rate was done in 2002, prior to the integration of Ilha Josina Machel in CISM’s health demographic surveillance system (HDSS). The study included 288 children aged 6–18 months, who were followed up by active detection of *P. falciparum* infection during 6 weeks after curative therapy with sulfadoxine-pyrimethamine (SP). Eighty-seven percent of participants had been re-infected after 6 weeks, with an incidence rate of malaria infection of 4.35 episodes per child year at risk (95 % CI 3.34–5.69) (unpublished data).

IRS started in 2004 and was implemented once every year, except for 2011. IRS was conducted using carbamates (lambda-cyhalothrin) at first, and dichloro-diphenyl-trichloroethane (DDT) since December 2006. IRS coverage levels in the area are unknown, however cross-sectional data from different surveys conducted by CISM since 2010 indicate that coverage of IRS in the previous 12 months in Ilha Josina Machel was 88 % in 2010, 70 % in 2011 and 2012, and 57 % in 2013 (unpublished data). Focal distribution of long-lasting insecticide treated bed nets (LLINs) by the NMCP started in 2013, however they were available for purchase beforehand and were also distributed free of charge to pregnant women through antenatal clinics. CISM survey data show coverage of LLIN usage of 27 % in 2010, increasing approximately to 43 % between 2011 and 2013. Additionally, starting in 2006, pregnant women attending antenatal clinic were administered intermittent preventive treatment (IPTp) with SP from the second term onwards.

During this study period (July 2003–June 2013), first-line treatment for uncomplicated malaria was amodiaquine plus SP until September 2006, followed by ACT (initially artesunate plus SP and from 2009 onwards arthemether–lumefantrine, Coartem^®^).

### Data collection

Since 2002, a passive case detection system was established at the IJMHP to cover all outpatient visits of children younger than 15 years, as part of the morbidity surveillance system run by CISM in all other health facilities in the DSS area and described in detail elsewhere [9]. Briefly, a standardized questionnaire gathering personal and demographic data (including the DSS unique permanent identification number, linking each patient to their geo-positioned household) as well as clinical information regarding the current illness is completed for each child attending the outpatient clinic. A qualified clinician records the physical signs and symptoms and their duration as referred by the child and/or accompanying guardian. A malaria blood smear (thick and thin films on the same slide) is performed from a finger prick for all children with measured fever (axillary temperature ≥37.5 °C), a history of fever in the preceding 24 h, or paleness, and blood is also collected into a microcapillary for packed cell volume (PCV) determinations. Final diagnosis/es using ICD-10 coding and treatment are recorded on the questionnaire by the clinician upon discharge or referral. Children fulfilling severity criteria are referred to the Manhiça District Hospital, where they are managed accordingly. Malaria cases are treated according to national guidelines.

Thin and thick blood films were read to quantify parasitaemia in the CISM laboratory according to standard procedures. Blood films were air-dried, Giemsa-stained, and examined to quantify parasitaemia using a light microscope fitted with a 100× oil immersion lens and a 10× eyepiece. Slides were declared negative only after counting 2000 leukocytes. Parasite numbers were converted to a count/µL by assuming a standard leukocyte count of 8000/µL for blood slides collected until the end of June 2009 and using a fixed volume method [10, 11] from then onwards. Slides were first read at the health post using the ‘plus system’ [12] to guide case management. If the parasitaemia result was missing, results from the ‘plus’-reading were used instead for the malaria case definition. PCV was measured using a micro-haematocrit centrifuge and a Hawksley reader (Hawksley & Sons Ltd, Lancing, UK) to assess levels of anaemia among cases.

### Definitions

Clinical malaria was defined as the presence of fever (axillary temperature ≥37.5 °C) or history of fever in the preceding 24 h plus any asexual *P. falciparum* parasitaemia confirmed through blood smear microscopy. No age cut-off for the level of parasitaemia was used to increase the specificity of the definition, as this is the definition used at the outpatient clinic for all ages to diagnose and treat malaria.

The geometric mean parasite density (GMP) was calculated as the mean of the log-transformed parasite densities of clinical malaria cases per season. Mild anaemia was defined as a PCV between 25 and <33 %, moderate anaemia as a PCV of 15 to <25 % and severe anaemia as a PCV <15 %. Time was divided into ‘seasonal’ years (1 July–30 June of the following year) in order to capture the full transmission season and be able to account for any fluctuations of rain patterns or other environmental factors between years.

### Data management and analysis

Outpatient questionnaires were double-entered into a database using a program written in Fox Pro (Microsoft Corp, Seattle, WA, USA) at CISM. Statistical analyses were performed using Stata 13.1 (Stata Corp, College Station, TX, USA).

The health post-based positive rate of clinical malaria (number of clinical malaria cases divided by total outpatient visits to IJMHP) and slide positivity rates (number of positive blood smears divided by total number of blood smears collected) were calculated for every seasonal year from 2003–2004 to 2012–2013.

Yearly mean age differences were calculated for clinical malaria cases and non-cases. A difference in difference (DID) analysis was conducted to compare the shifts in ages in time between malaria cases and non-cases. Mean age differences between years for malaria cases and non-cases as well as the difference between those means were calculated using linear regressions with interaction terms between case category and season.

Yearly means of PCV and GMP by age group and their corresponding 95 % CI were also calculated to further explore the clinical and epidemiological trends of the disease through time. Analysis of variance (ANOVA) tests were conducted to compare means of normally distributed variables across seasonal years. PCV and GMPs were compared between age groups across the study period using multiple linear regressions and *t* tests. An increasing amount of outpatient visits with no PCV information was observed between 2007 and 2013 among clinical malaria cases, reaching 86 % of missing information for PCV among the malaria cases observed between 2012 and 2013. This was accounted for in the interpretation of results.

The DSS unique permanent identification number, which is recorded on the morbidity questionnaires when children attend the health post, was used to classify children as residents/non-residents in the study area. Minimum community-based incidence rates (MCBIRs) were calculated as the age-specific yearly number of malaria cases in children resident in Ilha Josina Machel who visited the outpatient clinic divided by the total child years at risk (CYAR) for that age group and year. CYARs were estimated from CISM’s DSS databases and children did not contribute to the numerator or denominator for an arbitrary period of 28 days after each episode of malaria. They were also excluded when they were outside the study area or after death. Clinical malaria incidence rate ratios (IRR) were calculated through Poisson regression to further explore the relative shift in clinical malaria incidence over the years within each age group. IRRs were also calculated for every age group separately, which permitted better characterization of the age-specific trends of the disease through time.

## Results

A total of 70,698 visits were conducted at the outpatient clinic of the IJMHP between 1 July 2003 and 30 June 2013. The number of annual visits steadily increased over the years, ranging from 5735 visits in 2003–2004 to 9851 visits in 2012–2013 (Table 1). The mean age of all paediatric visits steadily increased from 3.4 to 5 years during the study period. Females represented 51 % of the outpatient visits, and 36 % of the cases presented with fever at time of visit every year. Malaria testing rates were constantly above 95 % for any case with fever or who reported to have fever in the previous 24 h.

**Table 1:** Summary characteristics of clinical malaria cases attending the IJMHP between 2003 and 2013

Season	Total outpatient visits (%)	SPR (%)	Clinical malaria cases (%)	Mean age (SD)	Mean PCV (SD)	GMP parasites per µL (SD)
03–04	5735 (8)	2886 (55)	2940 (51)	2.9 (2.8)	29.7 (5.8)	5778.3 (2.4)
04–05	5213 (7)	3122 (66)	3189 (61)	3.0 (2.7)	29.0 (5.5)	5846.6 (2.4)
05–06	5660 (8)	3123 (63)	3202 (57)	3.4 (3.0)	29.2 (5.7)	5214.3 (2.7)
06–07	6474 (9)	2778 (51)	2813 (43)	3.7 (3.1)	30.3 (5.4)	5692.9 (2.6)
07–08	6152 (9)	1288 (27)	1354 (22)	4.0 (3.1)	30.6 (5.5)	9443.9 (2.4)
08–09	7366 (10)	1646 (28)	1709 (23)	4.5 (3.2)	31.2 (5.1)	9443.1 (2.3)
09–10	7289 (10)	2049 (37)	2214 (30)	4.8 (3.2)	30.7 (5.2)	14,951.3 (2.6)
10–11	8291 (12)	3328 (47)	3336 (40)	5.3 (3.5)	31.1 (5.3)	15,788.5 (2.7)
11–12	8667 (12)	3354 (46)	3454 (40)	5.2 (3.4)	31.7 (5.3)	12,424.6 (2.7)
12–13	9851 (14)	4742 (56)	4986 (51)	5.7 (3.5)	32.4 (5.3)^a^	17,316.0 (2.5)
Total	70,698 (100)	28,316 (48)	29,197 (41)	4.3 (0.01)	30.5 (0.03)	10,189.9 (1469.0)

*SPR* slide positivity rate, *SD* standard deviation, *PCV* packed cell volume, *GMP* geometric mean parasite density

^a^86 % clinical malaria cases without PCV information

**Fig. 3:**
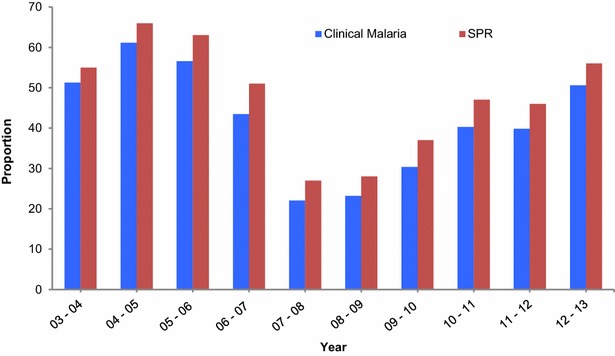
Yearly proportions of clinical malaria and SPR among children who attended the IJMHP (2003–2013)

More than half of all outpatient visits to the IJMHP between 2003 and 2006 were due to clinical malaria (51 % in the season of 2003–2004, 61 % in 2004–2005, and 57 % in 2005–2006). A sharp decline was observed between 2007 and 2009, when less than a quarter (23 %) of all visits to the health facility were associated with malaria. From 2009 onwards, the number of clinical malaria cases increased steadily reaching again 51 % of all outpatient visits in 2012–2013. A similar trend was observed when analysing slide positivity rates (SPRs) (Fig. 3).

**Fig. 4:**
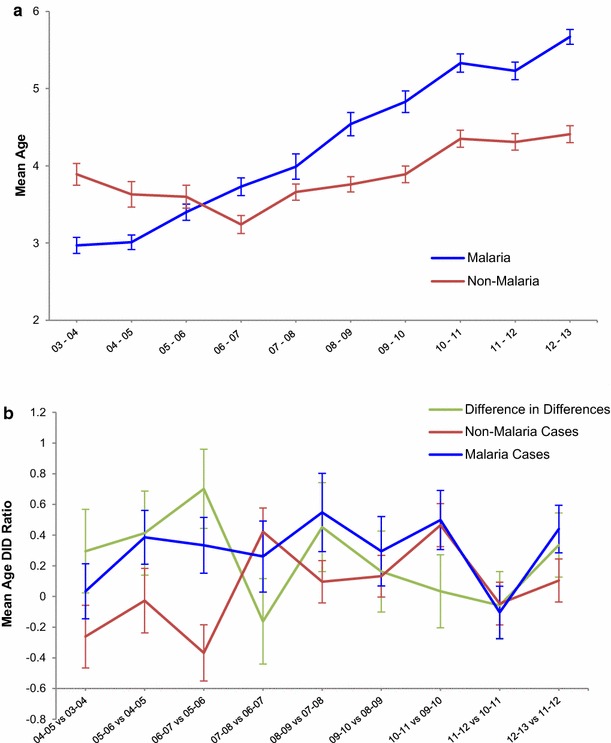
Mean age of malaria cases and non-malaria cases of the IJMHP from 2003 to 2013. **a** Mean age trends observed among children presenting at the IJMHP with clinical malaria (*blue*) or any other pathology (*red*) during the study time. **b** Presents the DID analysis that shows that the age difference from 1 year to the next between children with malaria (*blue*) was significantly larger than that for non-malaria cases (*red*). The *green line* represents the difference in mean age difference from 1 year to the next

The mean age of clinical malaria cases presenting to the IJMHP steadily increased for every seasonal year, shifting from 2.9 (SD 2.8) years in 2003–2004 to 5.7 (SD 3.5) year in 2012–2013. A different trend in mean age was observed among non-malaria cases, which was 3.9 (SD 3.8) in 2003–21004 and decreased to a minimum of 3.2 (SD 3.6) in 2006–2007, and increased steadily until 2012–2013, when it was 4.4 (SD 3.9) (Fig. 4a). The DID analysis to compare age shifting patterns between malaria cases and non-cases showed a significantly larger increase in the mean age of patients with malaria than those with other pathologies. In general, the increase in malaria case ages was significantly higher for every year compared to the previous one except for 2007–2008 compared to 2006–2007 and in between 2009 and 2012, where the increase in age was similar in both groups (Fig. 4b).

**Fig. 5:**
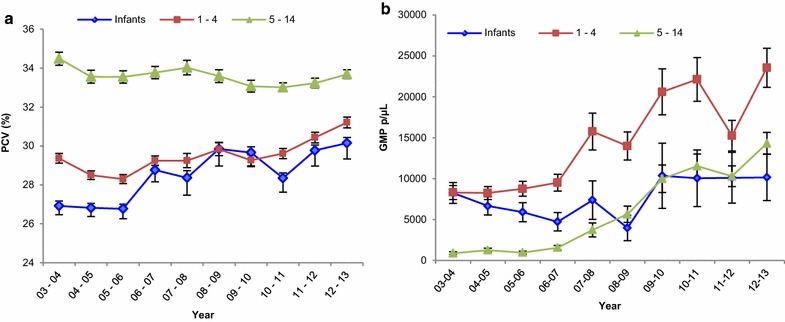
Yearly PCV and GMP tendencies by age group. **a** Yearly mean PCV of clinical malaria cases in infants (*blue*), 1–4 years olds (*red*), and 5–14 years olds (*green*). **b** Yearly GMP (parasites/µL) of clinical malaria cases in infants, 1–4, and 5–14 years olds, seen at the IJMHP

Out of all clinical malaria cases attending the IJMHP between 2003 and 2013, 46.8 % were mildly anaemic, 13.4 % moderately anaemic and 0.7 % suffered from severe anaemia. A significant increase in the mean PCV among clinical malaria cases was observed, shifting from 29.7 % (95 % CI 29.4–29.8) in 2003–2004, to 32.4 % (95 % CI 32.2–32.5, p < 0.0001) in 2012–2013. Figure 5a presents the changes in PCV levels by age group throughout the study period. Infants had a lower PCV compared to 1–4 and 5–14 year olds during the entire study period, with an increasing trend observed from 2003–2004 (mean PCV 26.9 %, 95 % CI 26.5–27.4) to 2012–2013 (mean PCV 30.2 %, 95 % CI 29.3–31.0).

GMPs among clinical malaria cases experienced a steady and significant increase from 5778 parasites/µL in 2002–2003 to 17,316 parasites/µL in 2012–2013 (p < 0.0001). Stratification by age group revealed that the overall shift in GMP was mainly driven by children older than 1 year who experienced infections of significantly higher GMPs every year, with the steepest increase in children of 5–14 years old. On the other hand, GMPs of infants were similar over time, with a minor drop in 2008–2009 (Fig. 5b).

The clinical malaria MCBIRs at the IJMHP experienced a similar pattern to that observed for the proportion of total visits with clinical malaria, starting with 667 cases per 1000 CYAR (95 % CI 639–697) in 2003–2004, and decreasing to 206 cases per 1000 CYAR (95 % CI 191–221) in 2007–2008. A resurgence was later observed, reaching a total of 871 cases per 1000 CYAR (95 % CI 840–903) in 2012–2013, the zenith of the documented incidence in the area. Table 2 presents the clinical malaria MCBIRs and IRRs for every year of the study overall and by age group. Overall, children aged 1–4 years experienced the highest number of incident clinical cases per 1000 CYAR at any point in time except for the first year, and children aged 5–14 years were the age group with the lowest incidence during the majority of the study period.

**Table 2:** Yearly clinical malaria MCBIRs (cases per 1000 CYAR) and incidence rate ratios in infants, 1–4, and 5–14 year olds, attending the IJMHP

Years	All		Infants		1–4 years old		5–14 years old	
	Rate (95 % CI)	IRR (95 % CI)	Rate (95 % CI)	IRR (95 % CI)	Rate (95 % CI)	IRR (95 % CI)	Rate (95 % CI)	IRR (95 % CI)
03–04	667.8 (639.5–697.3)	1	1643.9 (1499.0–1802.9)	1	1288.6 (1219.2–1362)	1	191.4 (172.4–212.6)	1
04–05	713.2 (684.2–743.4)	1.1 (1.0–1.1)	1532.3 (1391.0–1688.1)	0.9 (0.8–1.1)	1475.5 (1401.6–1553.3)	1.1 (1.1–1.2)	195.2 (176.2–216.2)	1.0 (0.9–1.2)
05–06	621.7 (595.12–649.5)	0.9 (0.9–1.0)	1109.8 (994.4–1238.5)	0.7 (0.6–0.8)	1245.2 (1178.1–1316.1)	1.0 (0.9–1.1)	224 (203.8–246.2)	1.2 (1.1–1.3)
06–07	483.8 (461–507.8)	0.7 (0.7–0.8)	649.8 (566.3–745.6)	0.4 (0.3–0.5)	992.2 (933.3–1054.8)	0.8 (0.7–0.8)	201 (182.4–221.4)	1.1 (0.9–1.2)
07–08	206 (191.6–221.5)	0.3 (0.3–0.3)	217.6 (171.8–275.5)	0.1 (0.1–0.2)	385.7 (350.4–424.4)	0.3 (0.3–0.3)	112 (98.6–127.3)	0.6 (0.5–0.7)
08–09	281.5 (264.7–299.4)	0.4 (0.4–0.5)	200.4 (156.6–256.6)	0.1 (0.1–0.2)	556.8 (514–603.2)	0.4 (0.4–0.5)	158.2 (142.4–175.8)	0.8 (0.7–1.0)
09–10	369.8 (350.5–390.2)	0.5 (0.5–0.6)	254.3 (204.8–315.8)	0.2 (0.1–0.2)	690.7 (643–741.9)	0.5 (0.5–0.6)	228 (208.9–248.9)	1.2 (1.1–1.4)
10–11	638.5 (612.8–665.3)	0.9 (0.9–1.0)	369.19 (304.9–447)	0.2 (0.2–0.3)	1040.6 (981.3–1103.5)	0.8 (0.7–0.9)	476.88 (448.8–506.7)	2.5 (2.2–2.8)
11–12	677.8 (651–705.9)	1.1 (1.0–1.1)	525.7 (446.5–619)	0.3 (0.3–0.4)	1106.4 (1042.5–1174.2)	0.9 (0.8–0.9)	505.2 (476.3–535.8)	2.6 (2.3–2.9)
12–13	871.4 (840.5–903.5)	1.4 (1.3–1.5)	544.4 (463.7–639.2)	0.3 (0.3–0.4)	1290.2 (1219.5–1365.1)	1.0 (0.9–1.1)	731.3 (696.1–768.3)	3.8 (3.4–4.3)

**Fig. 6:**
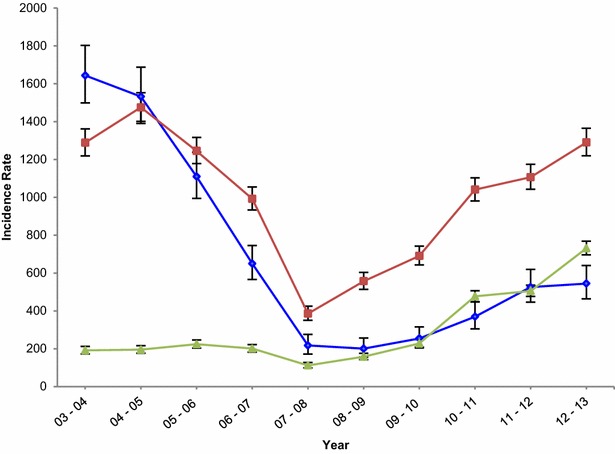
Yearly incidence rates (in 1000 per CYAR) by age group, observed at the IJMHP. Yearly MCBIRs in 1000 per CYAR were calculated to assess clinical malaria incidence observed in infants (*blue*), 1–4 years olds (*red*), and 5–14 years olds (*green*), at the IJMHP between 2003 and 2013. CYARs were obtained from CISM’s DSS, which is updated on a yearly basis

Clinical malaria MCBIR trends varied significantly between different age groups throughout the study period. Incidence rates among infants consistently decreased to a third of the baseline levels by 2012–2013 (RR = 0.3, 95 % CI 0.3–0.4, p < 0.001). One to 4 years olds also experienced a 70 % reduction in the rate of clinical malaria cases between 2003–2004 and 2007–2008 (RR = 0.3, 95 % CI 0.3–0.3), although this was not maintained, and an increasing trend was later observed, reaching similar incidence levels to those of 2003–2004 in 2012–2013 (RR = 1.0, 95 % CI 0.9–1.0). Finally, clinical malaria cases per 1000 CYAR among children older than 5 years remained unchanged between 2002 and 2009, and experienced a significant increase until 2013. By the last year of the study period, the incidence among children older than 5 years was 3.8 (95 % CI 3.4–4.3) times higher than it was in 2003–2004 (Fig. 6).

## Discussion

This study aimed to characterize the epidemiological trends of clinical malaria observed over a period of 10 years at a rural health centre in an area that had a high malaria transmission in 2002 and experienced a drop in malaria cases followed by a subsequent resurgence. IJMHP experienced a drop in clinical malaria cases between 2004–2005 and 2007–2008 followed by a resurgence of cases observed until the end of the study period in 2012–2013, detecting similar clinical malaria positive rates in 2012–2013 as that observed in 2003–2004. These patterns resemble those observed in the rest of Mozambique, that despite increasing availability of funding for malaria control, has experienced an increase in the number of malaria cases reported to the NMCP since 2009 after a previous drop of cases [1]. Nevertheless, the national health management information system does not yet offer complete and accurate data that allows further characterization of the profile of malaria cases observed nationally, and data from Malaria Indicator Surveys are outdated (last performed in 2007).

In Ilha Josina Machel, a sharper increase of clinical cases was observed particularly among children on their first to fifth birthday between 2007 and 2013, as well as those between the ages of 5 and 15 years. Overall, a shift in the age distribution of the clinical malaria cases towards older children was observed particularly among malaria cases, indicative of an alteration in the transmission intensity patterns in the area [13] and a potential consequent impact on the naturally acquired immunity (NAI) of the population.

The current analysis does not allow for any assessment of causality to evaluate which factors contributed to the decline and subsequent rise in transmission in the area. Rainfall patterns during the study period show a decrease in precipitation levels around the same years as malaria cases started to drop. This suggests that environmental factors may have had a considerable effect on the epidemiology of malaria in the past decades. Further spatiotemporal studies in the area shall be conducted to more accurately measure the impact of climatic factors on malaria through time. However, it is plausible that the introduction of new malaria control measures also played an important role in the initial decline in transmission. IRS was first deployed in Ilha Josina Machel in 2004–2005, according to district records, which correlates with the observed decrease in incidence between 2006 and 2009. However, an increase in the number of clinical malaria cases was later observed, which could be associated with a decrease in the IRS effectiveness due to low coverage (as reported between 2010 and 2013), or lack of continuity of IRS activities (such as in 2011). In fact, malaria resurgences have been observed in a number of different settings across the world since the beginning of the twentieth century, most of which are attributed to weakening of malaria control programmes due to resource constraints, and a subsequent inability to ensure continuity of IRS in places where it had already been delivered [14].

The sudden drop in the number of clinical malaria cases between 2007 and 2009 possibly correlates with a consequent drop in transmission intensity of malaria in the area at that time, considering that no changes were made in the reporting system and that malaria-testing rates remained constant throughout this period. The significant shift in the age distribution of clinical malaria cases towards older children observed in IJMHP supports this hypothesis, as in lower transmission settings young children will be exposed to less infectious bites and will take longer to build up a naturally acquired immunity, with older children having a relatively high incidence of clinical malaria [15, 16]. Unpublished entomological data from Ilha Josina of 2006 and 2007 further support this drop in transmission, showing an entomological inoculation rate (EIR) of 77 infectious bites per person during the dry season of 2006, to an EIR of 0.8 during the dry season of 2007. Similar shifts in the age distribution of clinical malaria cases as a function of transmission intensity have been extensively reported in the past in other sub-Saharan African countries [17–21].

The low-transmission period between 2007 and 2009 was followed by an immediate increase in the incidence of clinical malaria cases, particularly among 1–4 years olds and later also in the 5–14 years olds. During the lower malaria transmission period there was a decrease in the exposure to parasites in children (evident in the incidence rates among infants since 2007), delaying the acquisition of NAI in younger children and diminishing the premunition in older children that had already developed some NAI during the previous years [22]. Several studies, conducted to evaluate this question through actively interrupting exposure to the parasite using chemoprophylaxis at a very young age, have reported a similar rebound effect in the incidence of clinical malaria cases among children in the treatment arm, up to 1 years after treatment [15, 23–25]. The biological mechanisms driving the acquisition of NAI are still not well understood; however, evidence from mathematical models [26, 27] and immunological studies [28, 29] clearly identify age, exposure to the parasite and transmission intensity as important factors involved in this process.

The lower increase in the incidence rate of clinical malaria cases among infants may be partly explained by a drop in transmission; however other interventions such as the introduction of vector control activities targeting pregnant women and infants could have also contributed to an increased protection against parasite exposure among this age group.

The age-specific GMP trends observed in this study, with significant increases in parasitaemia in children older than 1 year after 2007 also show a slower acquisition of NAI with age and a lower ‘anti-parasite’ immunity [22]. In addition, the increase of mean PCV with age despite a raise in parasitaemia could possibly be a reflection of lower chronic infections or lower number of episodes per year, which cause less anaemia and does not allow the anti-parasite immunity to develop fast, resulting in the delay in the rate of NAI and the observed high parasitaemic infections.

Insecticide resistance may also be a contributing factor to the observed resurgence in the number of clinical malaria cases in the area. Mosquito resistance to pyrethroids was first reported in 2011 in a nearby sugar cane plantation of Maragra in the district of Manhiça [30]. Another study in a Northern province also reported wide resistance to pyrethroids and carbamates in 2007 [31]. Entomological data from CISM further supports these findings revealing less than 9.6 % sensitivity to pyrethroids among the *A. funestus* population of the district of Manhiça [32]. Insecticide resistance to pyrethroids has serious implications regarding the likely impact of LLINs towards reducing malaria transmission in the area, which since 2011 may have contributed to the increasing malaria incidence trends in the area.

The estimates obtained from this analysis are subject to a series of limitations. First, access to health care may have increased through the years. This could have caused an underestimation of the malaria burden in the area on the first years of the study, and a consequent overestimation of IRRs when comparing rates across years. Second, IJMHP data are also susceptible to the Hawthorne effect [16], as the presence of CISM, its impact on case management as well as research studies conducted, may affect the overall epidemiology of malaria in the study area of Manhiça. Particularly, Ilha Josina Machel was included as one of the sites where the RTS,S vaccine trials took place between 2002 and 2008. This analysis was also affected by missing data of PCV particularly in the last years of the study, which may have affected the estimates of severe and mild anaemia cases during these years.

Additionally, this study aims to provide a temporal description of the clinical malaria epidemiology in a health facility. Therefore, it did not conduct any sociodemographic or environmental risk factor analysis, which would enrich the interpretations withdrawn from the results obtained from this particular assessment. Separate studies should be conducted to respond to the hypothesis raised in this study, identify the main drivers of malaria transmission in the area and determine the social factors that may put a resident in the area at a higher risk of malaria than others.

## Conclusion

This study shows a reduction in the clinical malaria incidence in a small area of initially high transmission in Southern Mozambique, and a subsequent resurgence of cases. Evidence from this study indicates that efforts to control malaria and reduce transmission need to be sustained over time in order to guarantee gains in the long term. Malaria control programmes with limited resources, such as the one in Mozambique, should consider malaria control interventions that ensure sustainability and target the most vulnerable populations, particularly infants and children under 5, at all times. Considering the costs and operational difficulty of this task, NMCPs should consider investing in innovative strategies to drastically interrupt transmission and eliminate the parasite reservoir from communities in a short period of time, and subsequently concentrate efforts to manage and eliminate remaining foci of transmission and imported infections.

## Abbreviations

ACT: artemisinin-based combination therapy
ANOVA: analysis of variance
CI: confidence intervals
CISM: Centro de Investigação em Saúde de Manhiça
CYAR: child years at risk
DID: difference in differences
EIR: entomological inoculation rate
GMP: geometric mean parasitaemia
HDSS: health demographic surveillance system
IJMHP: Ilha Josina Machel Health Post
IRR: incidence rate ratio
IRS: indoor residual spraying
ITNs: insecticide-treated nets
IPTp: intermittent preventative treatment for pregnant women
LLINs: long-lasting insecticide-treated nets
LSDI: lubombo spatial development initiative
MCBIRs: minimum community-based incidence rates
MDGs: Millennium Development Goals
NMCP: National Malaria Control Programme
NAI: naturally acquired immunity
PCV: packed cell volume
SP: sulfadoxine–pyrimethamine
SPR: slide positivity rate
